# Bull’s-Eye for Athletes (BEA): a measure of values-based behavior in sport and a psychometric evaluation using Rasch analysis

**DOI:** 10.1038/s41598-026-50333-4

**Published:** 2026-04-24

**Authors:** Gustaf Reinebo, Magnus Johansson, Markus Jansson-Fröjmark, Alexander Wiklund, Gertrud Ekman Öhrn, Thomas Parling, Alexander Rozental, Gerhard Andersson, Tobias Lundgren

**Affiliations:** 1 Centre for Psychiatry Research, Department of Clinical Neuroscience, Karolinska Institutet, & Stockholm Health Care Services, Region Stockholm, Norra Stationsgatan 69, Stockholm, SE-113 64 Sweden; 2 RISE Research Institutes of Sweden, Division Built Environment, System Transition, Stockholm, Sweden; 3 Department of Psychology, Stockholm University, Stockholm, SE-10691 Sweden; 4 Mottagningen för Elitidrott och hälsa, Riksidrottsförbundet and Stockholm Health Care Services, Region Stockholm, Riddargatan 1, Stockholm, Sweden; 5 Department of Health, Education and Technology, Luleå University of Technology, Luleå, Sweden; 6 Department of Behavioural Sciences and Learning, Department of Biomedical and Clinical Sciences, Linköping University, Linköping, Sweden

**Keywords:** Sports psychology, Values, Values-based behavior, Psychometrics, Rasch analysis, Health care, Psychology, Psychology

## Abstract

Working with values and values-based behavioral change in psychological interventions for athletes has become more common in sport psychology, but measures adapted for the sport context are needed. A new instrument based on the Bull’s-Eye Values Survey, called Bull’s-Eye for Athletes (BEA), was developed and adapted to assess values and values-based behavior in athletes. BEA consists of four values items, (1) Competition, (2) Training, (3) Preparation and recovery, and (4) Life outside of sport. Athletes identify their values and rate their behavioral commitment for each value item and to what extent they experience obstacles behaving according to their values, both related to their sport and life outside of sport. BEA was completed online by 155 athletes in Sweden competing at junior elite to senior international level. Scale dimensionality, invariance, monotonicity of response categories, and reliability were investigated using Rasch analysis. Convergent and discriminant validity was also investigated through correlation with other scales. Results indicated that BEA worked as a unidimensional scale with satisfactory psychometric properties. Item probability functions were investigated, and response categories were merged to achieve ordered item thresholds. No indication of invariance was found through differential item functioning (DIF) analyses for demographic (age, sex) or sport-related variables (competitive level, sport type, or current injury), nor for temporal stability of item properties through test-retest. Convergent and discriminant validity was supported by medium correlations with psychological inflexibility in athletes, general valued living, life satisfaction, subjective performance, and a few subtypes of performance anxiety and sport motivation. BEA is a promising instrument for measuring and working with values and values-based behavior in sport. Response categories were merged for two items (competition and training) to achieve ordered item thresholds. Labeling of the instrument’s response categories could be a potential improvement for BEA to be investigated. Further investigations are needed across various sports contexts with larger sample sizes, as well as evaluations of its usefulness in longitudinal designs in sport psychological intervention research as a measure of self-reported behavioral change.

**Supplementary Information:**

The online version contains supplementary material available at10.1038/s41598-026-50333-4.

## Background

With developments in clinical psychological intervention research during the 1990s and early 2000s, several new approaches within the cognitive and behavioral therapy (CBT) domain emphasized the importance of values and valued living as part of a healthy life. This period of CBT development is sometimes referred to as the “third wave” of CBT^1^. One psychotherapy approach that was developed during this period is Acceptance and Commitment Therapy (ACT)^2^. ACT is part of the behavioral tradition and focuses on helping clients to make behavioral changes by fostering present-moment awareness, acceptance toward thoughts and feelings, and clarification of what is important and meaningful in life and to behaviorally pursue those chosen values.

With psychological models emphasizing life values and related behavioral change, it became important to develop scales that measure such psychological processes^3^. Several scales have been developed over the years, with some focusing on values and valued living in general^4^. Other scales are life-domain specific^5^, and some are tailored for a specific context or targeting specific diagnostic groups, such as patients with chronic pain^6^.

In the wake of the developments in clinical psychology with interventions such as ACT, sport psychological interventions were soon influenced and started to apply mindfulness, acceptance, and values interventions with athletes^7^. Such intervention protocols, often referred to as Mindfulness- and Acceptance (MA) based approaches, are now commonly used with athletes worldwide. Systematic reviews and meta-analyses have shown that such studies have demonstrated effects on both psychological variables such as mindfulness^8^ as well as sport performance^9^, although several of the studies lack in study quality and further trials are needed using robust research designs to confirm the effects.

Although there are intervention protocols in sport psychology that emphasize the use of values-related interventions, the psychological processes of values and meaning in athletes have only been partly investigated. Athlete experiences of meaning and purpose in sport have been studied both qualitatively and quantitatively regarding, for example, sports’ contribution to meaning in life, personal growth through facing adversities in sport, and meaning in life outside the sport for athletes^10^. Instruments assessing such psychological processes in athletes are scarce, although the Meaning in Sport Questionnaire (MSQ) is one exception. MSQ is a nine-item measure of sport-specific meaning and purpose in athletes. It consists of two sub-scales: presence for meaning in sport and search for meaning in sport. MSQ showed satisfactory psychometric properties in an initial evaluation of the scale^11^. MSQ is a traditional survey and does not include a framework for working with values-based behavioral change – such an instrument is yet to be developed.

The Bull’s-Eye Values Survey (BEVS) is a clinical tool and measure developed in clinical psychology that includes: (1) values clarification and behavioral ratings on a dartboard to what extent these values are behaviorally pursued, (2) investigation and rating of obstacles to the valued living, and (3) a plan for behavioral change^5^. BEVS was developed as both a measure and behavioral change tool in ACT, with its theoretical basis in behavior analysis and relational frame theory (RFT), a behavioral account of human language and cognition^12^. RFT gives, as a behavioral theory, a functional understanding of values as verbally established motivation^13^, or more specifically as “freely chosen, verbally constructed consequences of ongoing, dynamic, evolving patterns of activity, which establish predominant reinforcers for that activity that are intrinsic in engagement in the valued behavioral pattern itself”^14^. In ACT, values-consistent behavior is seen as fundamental to a psychologically healthy life, and clients are encouraged to clarify and engage in what is meaningful and important to them in various life domains (e.g., relationships, education/career, hobbies, spirituality) to contact reinforcers in related activities and build healthy behavioral patterns of living^13^.

The purpose of the current study was to develop, adapt, and psychometrically evaluate a version of Bull’s-Eye for Athletes (BEA), a sport psychology instrument for measurement and therapeutic work with values and values-based behavior in relevant domains for the athletic endeavor and life. A modern test theory approach using Rasch measurement theory was applied as the main analysis of BEA. Rasch analysis enables an expanded examination of measurement functioning that provides information regarding both item functioning and person parameters to investigate aspects of validity and reliability^15,16^. More specifically, psychometric properties regarding unidimensionality, local independence, invariance, and monotonicity of response categories were examined. The construct validity of BEA was also investigated by correlation. It was hypothesized that convergent validity would be demonstrated between BEA and values in general life, life satisfaction, sport motivation, and subjective performance ratings. Further, it was hypothesized that discriminant validity would be demonstrated between BEA and sport-related psychological inflexibility, and performance anxiety.

## Methods

### Ethical considerations

This study was conducted in accordance with the Declaration of Helsinki and approved by the Swedish Ethical Review Authority (original reference number 2018/2344-31; amendment reference number 2019 − 01175). Participants gave their informed consent digitally on the web platform of the study. Part of the data used in this study was collected in association with an intervention study^17^ which was covered by the same ethical approval.

## Procedure

### Development of the Bull’s-Eye for Athletes instrument

The original Bull’s-Eye values survey^5^ served as the starting point for developing Bull’s-Eye for Athletes (BEA). The original Bull’s-Eye consists of three parts. In the first part, the respondent is asked to identify their values related to four life domains (work/education, leisure, personal growth/health, and relationships). Then the respondent marks, on a dartboard, to what extent he/she has lived in accordance with the identified values during the last two weeks. The dartboard is a Likert scale from 1 to 7, and a label on the outer rim of the dartboard (1) “My life is far from how I want it to be” (stands for “not living in line with my values at all”), and a label in the bull’s eye (7) “My life is just as I want it to be” (stands for “living fully in line with my values”). No labels are used for the response categories in between. In the second part, the respondent is asked to identify what obstacles hinder him/her from living in accordance with the valued direction and then rates on a scale from 1 (“doesn’t prevent me at all”) to 7 (“prevents me completely”), with labels only on the endpoints of the response categories (1 and 7). In the third part, the respondent identifies behavioral actions to take further steps in a valued direction that would bring them closer to the Bull’s-Eye.

The first author (GR) made the primary revisions for the development of BEA, with feedback from the last author (TL). BEA consists of three steps, just like the original Bull’s-Eye. For the first step (identifying values and dartboard ratings), the initial idea was to create domains that were both relevant to athletes’ sports endeavors and lives in general and broad enough for each individual athlete to describe their values without being constrained by the domain categories. These four domains/items are “Competition”, “Training”, “Preparation and recovery”, and “Life outside of sport”. In the first part, the respondent is asked to identify their values within each of these domains and then rate on the dartboard to what extent they have behaved in accordance with their chosen values in each domain during the past week. There are labels only on the outer rim (“I have not acted at all as I wanted to over the past week”) and in the Bull’s-Eye (“I have acted exactly as I wanted to over the past week”), with no labels on the response categories (rims) in between. In the web version of the dartboard used in this study, all seven rims were also marked with each respective rating score of 1 to 7. In the second part, the respondent is asked to identify what obstacles prevent her/him from behaving in line with their chosen values and then rate on a scale of 1 to 7 to what extent they are prevented by their described obstacles. Labels are only at the endpoints of the response categories (1 – “not at all prevented”, and 7 – “completely prevented”). Two separate ratings are done regarding obstacles, one in relation to their sport endeavor, and another in relation to their life outside sport. In the third step, the respondent is asked to identify and plan for behavioral actions in line with their chosen values in each BEA domain.

BEA was sent to three experienced sport psychology consultants to provide reflections regarding the instrument. This was to get initial content and face validity feedback from practitioners’ points of view. They were asked to give general input on the instrument and to reflect on whether the four value domains were comprehensive enough, or if the value domain definitions potentially left out important aspects of an athlete’s endeavor or life. Provided feedback served as initial support for the instrument’s content validity.

Only the original Swedish version of the BEA instrument was used in this study and is presented in Supplementary file 1. An English version was also translated by the first author to facilitate transparency and future use and is presented in Supplementary file 2.

### Participants

Athletes from various types of sports were eligible for this study. Therefore, a wide range of sports organizations and clubs that represent, organize, or participate in high-level sports, were contacted to be invited to participate or to help spread the invitation onward to potentially interested participants. Recruitment attempts were also made through social media (Facebook). 122 athletes in Sweden were recruited. In addition, data from 33 ice hockey players who had completed BEA at the beginning of an internet-based psychological intervention study^17^ were also included. Since they had completed BEA at the beginning of module 1 as among the first exercises of the intervention, the data was considered comparable enough and eligible to be included.

The aim was to primarily target athletes on an elite level, and the performance level of the study sample was categorized according to the geographical criteria of the current competitive level, which is a key criterion in sport performance level taxonomies^18,19^. The current sample corresponded to the following competitive categorizations: sectional level (competes against participants/teams from a larger part of the country but not from the entire country), national level (competes against participants/teams from all over the country), international level (competes against participants/teams from other countries, e.g., national teams), and junior elite (if there is an indication that competition is only at the junior level, ‘junior elite’ is used, and does not refer to a geographical categorization). In total, 155 athletes from junior elite to international level participated. One athlete did not complete the values ratings although other parts of BEA, but the participant was kept in the sample since the ratings of the other scales were completed and therefore contributed to the evaluation of those instruments in this study. Please see Table 1 for sample characteristics.

**Table 1:** Participant characteristics.

**Age**	
Mean	23.14
Standard deviation	4.78
Range	15–50
**Sex**	
Females	65 (42%)
Males	90 (58%)
**Currently injured**	
Yes	30 (19%)
No	125 (81%)
**Pursuing elite level commitment**	
Yes	144 (93%)
No	11 (7%)
**Sport type**	
Team sport	136 (88%)
Individual sport	19 (12%)
**Performance level**	
Junior elite	7 (5%)
Sectional	56 (36%)
National	71 (46%)
International	21 (14%)
**Sports represented**	
Soccer	60 (39%)
Ice hockey	43 (28%)
Floorball	12 (8%)
Volleyball	10 (6%)
Wrestling	8 (5%)
Synchronized swimming	5 (3%)
Swimming	3 (2%)
Tennis	3 (2%)
Basketball	2 (1%)
Handball	2 (1%)
Bowling	2 (1%)
Obstacle racing	1 (0.5%)
Badminton	1 (0.5%)
Discus	1 (0.5%)
Boxing	1 (0.5%)
Water polo	1 (0.5%)

Total *N* = 155.

### Other measures

Besides BEA, the following scales were administered to participants to enable convergent and discriminant validity investigations by correlation.

#### Psychological inflexibility in athletes

The Psychological Flexibility in Sport Scale (PFSS) is a seven-item scale that measures psychological inflexibility and experiential avoidance in athletes. The Swedish version has been evaluated using a factor analysis framework that indicated it to be a unidimensional scale with acceptable model fit in a confirmatory factor analysis (CFA) using Maximum Likelihood (ML) estimation based on a sample of 252 young athletes (mean age 17), χ^2^ = 14.99 (*p* = .183), CFI = 0.995, TLI = 0.991, RMSEA = 0.038, SRMR = 0.048 ^20^. A Persian version of the PFSS has also been evaluated using factor analysis, which further supported the use of the seven-item version^21^. Items are sport generic and negatively worded and focus on the athlete’s ability to handle thoughts and feelings during performance, such as “When I am competing my thoughts impair my performance”. Higher PFSS scores correspond to higher psychological inflexibility/experiential avoidance. All seven response categories had labels from “Never true” to “Always true”. The PFSS has to our knowledge not undergone Rasch or Item Response Theory-based analysis priorly. A Rasch analysis was performed for PFSS in the current study and reliability for the adjusted version of the scale had a sample Personal Separation Index (PSI, items capacity to separate participants on a latent continuum) of 0.89 on a scale from 0 to 1, and the Relative Measurement Uncertainty (RMU) was 0.90 (95% HDCI [0.868, 0.921]).

#### Life satisfaction

The Satisfaction With Life Scale (SWLS) measures global life satisfaction and respondents are asked to rate to what extent they agree to statements such as “I am satisfied with my life”. It is a five-item scale and did initially show satisfactory psychometric properties, such as high internal consistency (α = 0.87) and test-retest correlation coefficient of 0.82^22^. The unidimensional model of life satisfaction in SWLS has also been supported in a Swedish sample^23^. Prior Rasch analyses of the SWLS have raised issues with some aspects of the scale’s functioning, such as having too many response categories^24,25^. A higher score on SWLS corresponds to higher life satisfaction. The five items have seven scale steps that all had labels in this study from “completely disagree” to “completely agree”. A Rasch analysis was performed for SWLS in the current study and reliability for the adjusted version of the scale was RMU = 0.79 (95% HDCI [0.73, 0.84]), and sample PSI = 0.77.

#### Valued living

Valuing Questionnaire (VQ) was used to measure general valued living. It consists of ten items and has in previous validation shown to constitute two factors of five items each, (1) progress in valued living with items such as “I continued to get better at being the kind of person I want to be”, and (2) obstruction to valued living with items such as “I spent a lot of time thinking about the past or future, rather than being engaged in activities that mattered to me”. The items have seven response categories from 0 (“Not at all true”) to 6 (“Completely true”) with labels only at the endpoints of the Likert scale. Higher scores on the obstruction sub-scale correspond to higher levels of being obstructed to a valued living. Further, higher scores on the progress sub-scale correspond to higher levels of progress towards a valued living. The scale has been evaluated using a factor analysis paradigm that has shown good model fit for the two-factor solution, as well as concurrent validity with related measures, and good internal consistency (in a non-clinical sample Cronbach’s α was 0.87 for both the progress and obstruction sub-scale)^4^. The two-factor solution has also been supported in a Swedish context in patients with chronic pain^26^. To our knowledge, the scale has not undergone Rasch analysis previously. A Rasch analysis was performed for each sub-scale of VQ (called VQ progress, and VQ_obstruction) in the current study. Reliability for the adjusted version of the sub-scale VQ_progress had a sample PSI of 0.77, and RMU = 0.83 (95% HDCI [0.79, 0.87]). Reliability for the adjusted version of the VQ_obstruction had a sample PSI of 0.69, and RMU = 0.72 (95% HDCI [0.65, 0.79]).

#### Sport motivation

Sport Motivation Scale-2 (SMS-2) is a measure of sport motivation and is theoretically based on the self-determination theory of motivation. It was developed and evaluated using a factor analysis framework that showed good model fit for a six-factor solution for the 18-item instrument (3 items in each sub-scale) with acceptable internal consistency for each sub-scale ranging from 0.70 to 0.88^27^. This six-factor structure has been supported in subsequent studies in various cultural sports contexts^28–31^. The English version of the SMS-2 was translated into Swedish following a translation-back-translation procedure by two of the study authors, further calibrated by a third study author. All study authors involved in the translation are native Swedish speakers and fluent in English. Each sub-scale is related to a sub-type of sport motivation, see example items in brackets followed by reliability estimates (RMU, and sample PSI) for each respective sub-scale found in this study: intrinsic regulation ( “Because it gives me pleasure to learn more about my sport”), RMU = 0.71 (95% HDCI [0.64, 0.78]), sample PSI = 0.57; integrated regulation (“Because practicing sports reflects the essence of who I am”), RMU = 0.63 (95% HDCI [0.53, 0.71]), sample PSI = 0.53; identified regulation (“Because I found it is a good way to develop aspects of myself that I value”), RMU = 0.75 (95% HDCI [0.68, 0.81]), sample PSI = 0.66; introjected regulation (“Because I would feel bad about myself if I did not take the time to do it”), RMU = 0.25 (95% HDCI [0.08, 0.40]), sample PSI = 0.27; external regulation (“Because I think others would disapprove of me if I did not”), RMU = 0.61 (95% HDCI [0.52, 0.70]), sample PSI = 0.38; and amotivated regulation (“I used to have good reasons for doing sports, but now I am asking myself if I should continue”), RMU = 0.70 (95% HDCI [0.62, 0.77]), sample PSI = 0.5. All seven response categories have labels on a Likert-scale from “Does not correspond at all” to “Corresponds completely”. Higher scores on each sub-scale reflect higher levels for that sub-construct (no reversed scoring of items). To our knowledge, SMS-2 has not undergone Rasch analysis previously and was performed in this study, with separate Rasch analyses for each sub-scale. As each sub-scale only contains 3 items, analyses should be acknowledged as limited from the start, as bad-performing items will not be removed since a subsequent Rasch re-analysis containing only two items would not be meaningful.

#### Performance anxiety

Sport performance anxiety was measured with the Competitive State Anxiety Inventory-2 Revised (CSAI-2R). CSAI-2 was originally developed by Martens et al. ^32^ and revised by Cox et al.^33^. The scale has previously been evaluated in Swedish athletes and the results of the psychometric evaluation also supported the use of the revised version (CSAI-2R) as superior to the original version, demonstrating the most acceptable model fit in a CFA^34^. The revised version (CSAI-2R) was therefore applied in this study. It consists of 17 items that constitute three factors. See example items in brackets followed by reliability estimates (RMU, and sample PSI) for each respective sub-scale in this study: cognitive anxiety (“I am concerned about choking under pressure”), RMU = 0.76 (95% HDCI [0.70, 0.82]), sample PSI = 0.68; somatic anxiety (“My body feels tense”), RMU = 0.77 (95% HDCI [0.71, 0.83]), sample PSI = 0.6; and self-confidence (“I am confident about performing well”), RMU = 0.85 (95% HDCI [0.81, 0.88]), sample PSI = 0.8. All four response categories have labels from “Not at all” to “Very much so”. Higher scores on each sub-scale reflect higher levels for that sub-construct (no reversed scoring of items). CSAI-2R has, to our knowledge, not undergone Rasch analysis priorly which was performed in this study. Separate Rasch analyses were conducted for each sub-scale.

#### Subjective performance

Subjective performance was assessed using a single item asking the respondent to rate the following statement: “During the past week, my athletic performance has been”. The item was rated on a scale from 1 (“Extremely poor”) to 10 (“Extremely good”) with labels only on the endpoints of the scale (response categories 1 and 10).

### Data collection

A secure web platform called “iterapi”^35^ was used to collect the data. It has been extensively used in other research projects, primarily in studies investigating internet-based psychological interventions. It was also on this platform that the psychological intervention study on ice hockey players was conducted from which BEA data was used for the current study^17^.

Initially, a pilot testing of the test administration procedure was undertaken with six athletes for which two of the study authors were present (AW and GEÖ). After completion of BEA and the scales, respondents could evaluate the administration procedure and if there were any questions or difficulties regarding it. After the pilot testing, some modifications were made regarding the information about the administration procedure that the participants received.

Participants in the Bull’s-Eye for Athletes study registered on the web platform and gave their digitally informed consent to participate in the research. They were then sent a link to the first survey (demographical questions, PFSS, SWLS, VQ, SMS-2, CSAI-2R, and the subjective performance rating), and once completed, they were sent information on how to log on to the web platform (using two-step verification) to complete BEA. For technical reasons related to using the dartboard in BEA as part of rating the value items, it had to be done on the web platform as a subsequent step after completing the first survey. Two weeks after completing BEA they were asked to login and complete BEA once again as part of the test-retest data collection procedure. If the survey or BEA had not been completed, reminders were sent to the participants. The survey and BEA were completed digitally, via digital devices such as cell phones or computers. The data collection procedure was conducted between February and May 2019.

### Statistical analysis

BEA was psychometrically evaluated using Rasch analysis by assessing its measurement properties, including unidimensionality, invariance, local independence, and response category monotonicity^16,36^. The psychometric evaluation also included an investigation of construct validity by correlation with other constructs.

Dimensionality was assessed in multiple ways. Conditional item fit^37^ was investigated by information-weighted mean square statistics (infit MSQ) using simulation (parametric bootstrapping) to estimate cutoff values with 100 bootstrap iterations as suggested for samples with < 250 participants^38^. Item-restscore analysis was undertaken which is another way to investigate whether items fit the Rasch model, by comparing observed and expected associations between each item and a score based on the remaining items using Goodman and Kruskal’s 𝛾^39^. Potential multidimensionality or clusters in data were also visually inspected by plotting the first residual contrast loadings based on a PCA of the standardized Rasch model residuals and item locations.

Local independence of items was investigated through residual correlations (Yen’s Q3) for each item-pair, using simulation-based cutoff values^40^. Local independence was also investigated by partial gamma coefficients^41^.

Response category monotonicity was examined by inspecting each item’s scale step probability distribution. Scale step thresholds are the locations on the latent continuum where two scale steps intersect and were inspected for being in order (in relation to the other scale steps and their place on the logit scale), and for being the most probable response category at some point on the latent continuum (otherwise the scale step does not provide any information). Response categories were merged when disordered or not being the most probable response category at any time on the latent continuum to achieve response category monotonicity. Targeting of items was investigated by comparing the parallel distributions of person and item threshold locations on the same logit scale and calculating the mean and standard deviation for each respective distribution.

Invariance was investigated by Conditional Likelihood Ratio (CLR) tests and Differential Item Functioning (DIF) analyses. CLR is a global test of fit that assesses homogeneity and whether item parameters are homogeneous across all levels of theta (*θ*) ^16^. DIF analyses investigate whether item locations are similar across sub-groups of respondents. DIF was investigated in relation to demographic variables (age, sex), sport variables (competitive level, sport type, and being injured), and high/low scorers on the scale. Test-retest was also investigated within the framework of DIF (item location stability over time), comparing item functioning between testing at two time points (planned as two weeks in between). Median days between test-retest was 16.80 days (IQR = 6.18 days). Test-retest was not investigated by correlation since it can result in a test of person properties stability over time rather than a test of item properties stability over time and should therefore not be considered a test of invariance^36^.

Reliability was investigated using Relative Measurement Uncertainty (RMU) with 95% highest density continuous intervals (HDCI). RMU is based on Bayesian measurement that estimate a posterior distribution of reliability and correlates two random draws from each subject’s posterior distribution, which will indicate the uncertainty of the measurement^42^. RMU was estimated using Weighted Likelihood Estimation (WLE)^43^ and Metropolis-Hastings sampling of plausible values. A point estimate of sample-based Person Separation Index (PSI, items capacity to separate participants on a latent continuum) will also be reported^44^.

Validity investigations by correlation with other scales were computed with complete cases (*n* = 120), by removing participants from the intervention study (*N* = 33)^17^ as they had not completed the other scales, 1 participant with missing BEA values, and 1 outlier respondent was also removed detected through visual inspection. Rasch analyses were also performed for the scales used in the validity analyses prior to the correlations with BEA, as described earlier under each measure. Sub-scales of multidimensional scales (VQ, SMS-2, CSAI-2R) were analyzed separately. Respondent’s latent *θ* values (“factor scores”), estimated with WLE^43^, were correlated with estimated *θ* values of BEA using Pearson’s product-moment correlation since *θ* is on a logit scale and therefore interval data. Partial correlations between BEA and the sub-scales within each multidimensional scale (VQ, SMS-2, and CSAI-2R respectively) were also computed to investigate the association between BEA *θ* and each sub-scale *θ* while controlling for the shared variance among the sub-scales from the same instrument. Reliability was investigated with RMU and PSI in the same way as BEA. Since the subjective performance rating was only one item, Rasch analysis could not be performed. It was therefore correlated with BEA using Spearman’s rank correlation since the ordinal scores are not interval data. Due to some participants not completing BEA and the other scales on the same day as intended, sensitivity analyses were conducted for all validity analyses. Results were compared with analyses based solely on data from participants who completed the other scales and BEA during the same day (defined as within 24 h, *N* = 103). A significance level of *p* < .05 was applied. Correlation strength was interpreted as small (*r* = .10 − .29), medium (*r* = .30 − .49), or large (*r* ≥ .50)^45^.

The statistical software R (version 4.4.2) and R studio (2024.12.0 + 467) were used to compute all analyses. Rasch analyses were conducted using Conditional Maximum Likelihood using the Partial Credit Model^46^. Tests for conditional item fit, loadings on the first residual contrast, residual correlations, as well as figures, were conducted using the easyRasch package version 0.3.4.1^47^, which in turn uses other R packages. CLR tests, item-restscores, and partial gamma coefficients were conducted using the iarm package version 0.4.3^48^, and DIF analyses were conducted using the psychotree package version 0.16.1^49^. Response categories were analyzed using the eRm package version 1.0–6^50^ and the mirt package version 1.43 ^51^, the latter package was also used to investigate reliability with RMU and sample PSI. Correlation analyses were conducted using the correlation package version 0.8.6^52^. Tables were created using the flextable package^53^. All code and analyses are available in an online supplementary analysis report (https://gustafre.github.io/Bulls-Eye-for-Athletes/) created with Quarto^54^.

## Results

### Rasch analysis of Bull’s-Eye for Athletes

#### Response category distributions

**Fig. 1:**
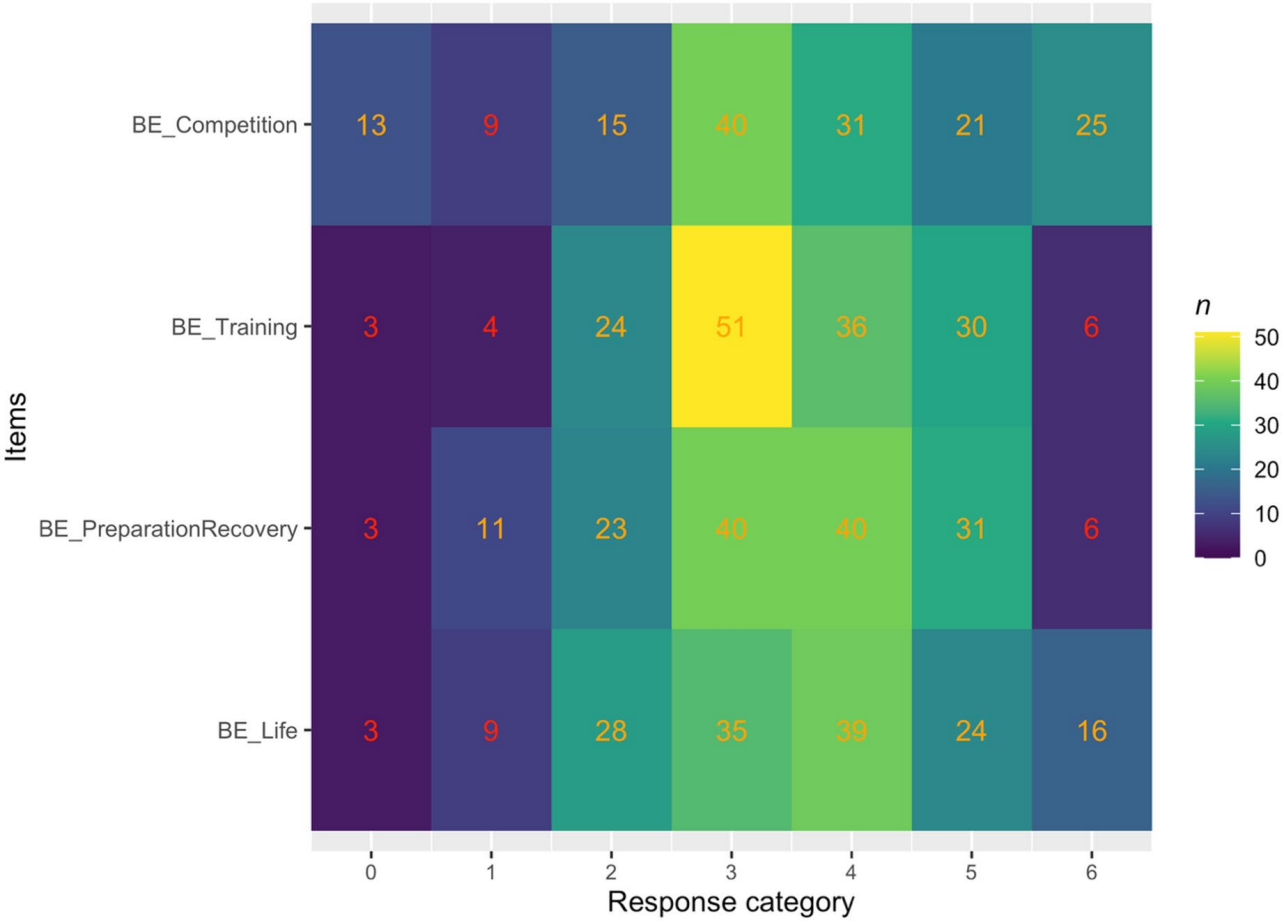
Distribution of responses for each response category for all BEA items.

Response category distributions are presented in Fig. 1. There are some indications that BE_Competition endpoints on the dartboard were endorsed to a larger extent than other items, which could indicate problems with item severity.

### Rating scale

**Fig. 2:**
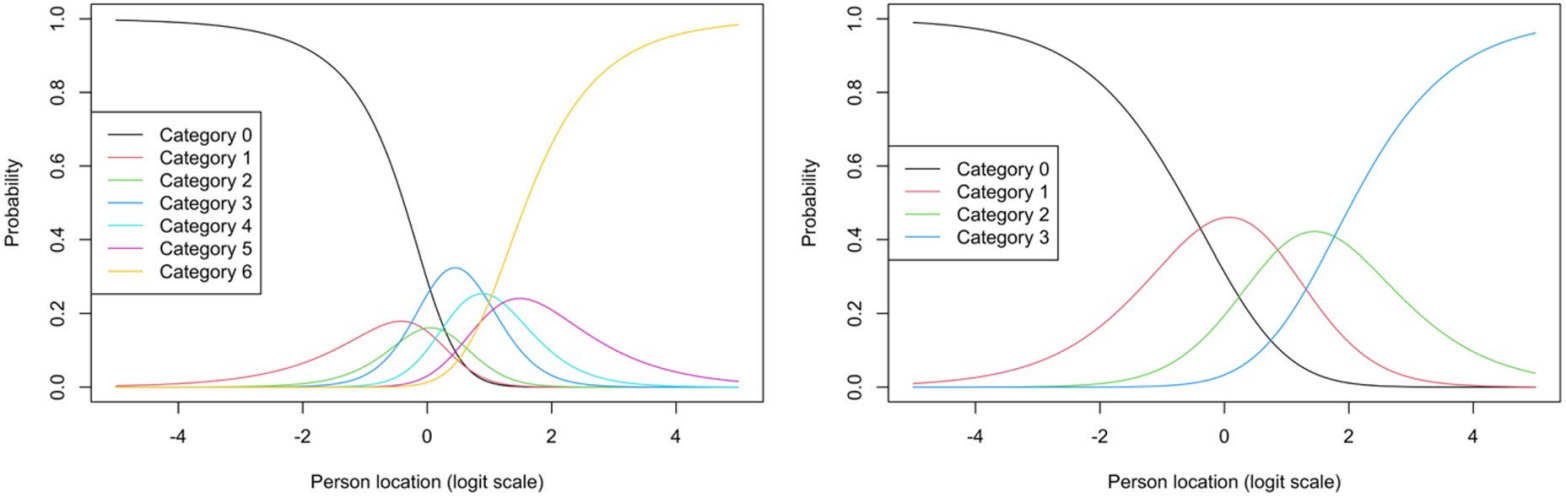
Item characteristic curves comparison for BE_Competition, original (left) and recoded (right) version. In the original item version (left), response categories 1, 2 and 5 are not the most probable response category (y-axis) at any point on the logit scale (x-axis) and are therefore merged with other response categories (1 with 0; 2 with 3; 5 with 4), which will also correct the disordered item thresholds.

**Fig. 3:**
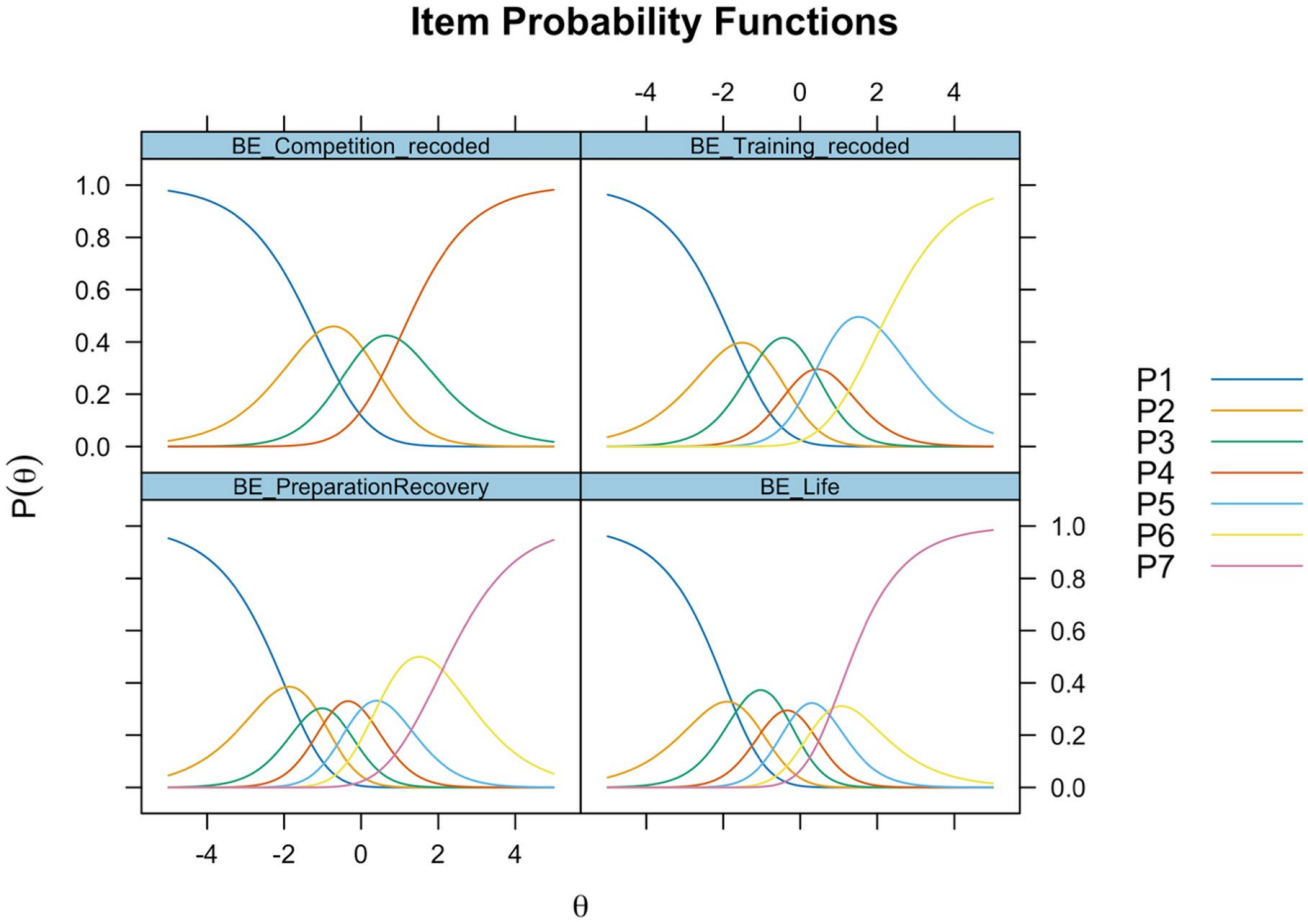
Item probability functions for each item. The probability for each response category is shown on the y-axis, given a respondent’s location on the latent construct (*θ*) on the x-axis.

Scale steps were merged due to not being the most probable response category at any point on the logit scale (*θ*), to make item thresholds ordered and achieve response category monotonicity. Disordered item thresholds were found for BE_Competition and BE_Training and were therefore re-coded (scale steps were merged). All items had some response categories with small distances between them. An example comparing item characteristic curves for original scale steps and merged scale steps is provided regarding BE_Competition, see Fig. 2. Original and recoded scale steps for all items (as well as item recoding structures) can be seen in the online supplementary analysis report. Recoded response category probabilities are presented for all BEA items in Fig. 3.

### Scale dimensionality

**Fig. 4:**
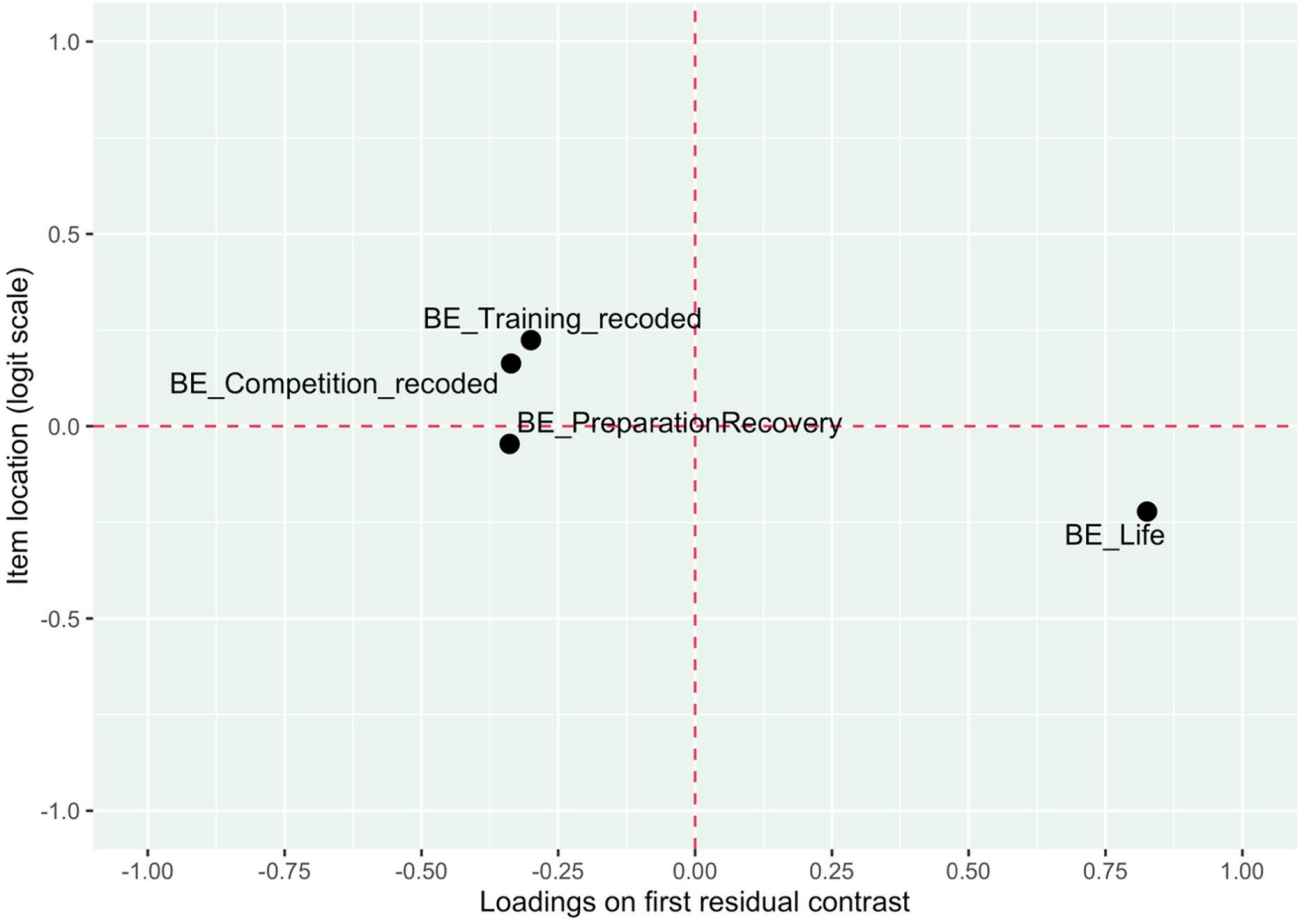
Item loadings on the first residual contrast factor.

The CLR tests of global fit yielded a non-significant result (CLR = 27.3, df = 19, *p* = .098), indicating that item parameters are homogeneous across all levels of *θ*. BE_Competition_recoded was indicated to be borderline overfit to the model and possibly redundant. BE_Life was also close but to the upper infit threshold, indicating it as potentially underfit to the model. However, both items’ infit were inside their thresholds. There were no further indications of misfitting items in the conditional item fit investigation (see Table 2). No misfitting items were further supported in the item-restscore analysis, which demonstrated no significant differences for any item. There were no item-pairs with residual correlations above the cutoff value. Nor were there any significant gamma coefficients indicating local dependence between any item pairs. This supports the assumption of local independence for all items. A minor indication of potential multidimensionality was found. Item locations and loadings on the first residual contrast factor indicate some clustering for the sport-related items in relation to the life outside of sport item (BE_Life), see Fig. 4.

**Table 2:** Conditional item fit.

Item	Infit MSQ	Infit thresholds	Infit difference	Relative item location
BE_Competition_recoded	0.829	(0.818, 1.179)	no misfit	-0.04
BE_Training_recoded	0.984	(0.805, 1.161)	no misfit	0.02
BE_PreparationRecovery	1.036	(0.807, 1.177)	no misfit	-0.25
BE_Life	1.172	(0.817, 1.183)	no misfit	-0.42

Note. Infit MSQ = conditional information weighted mean square statistics (*n* = 154 complete cases). Simulation-based thresholds from 92 simulated datasets.

### Differential item functioning and test-retest

There was no DIF found related to demographic variables such as sex (females *n* = 65, males *n* = 89) or age (≤ 23 years *n* = 84, compared to > 23 years *n* = 70). Nor was any DIF found related to sport variables such as competitive level (junior elite and sectional level *n* = 62, compared to national and international level *n* = 92), sport type (individual sport *n* = 19, team sport *n* = 135), or for currently being injured (injured, *n* = 29; not injured, *n* = 125). High and low scorers (split by median score) on BEA were also compared with no DIF detected. Test-retest was also investigated by DIF analysis with no significant difference found on BEA between testing at time point 1 (*n* = 154) and testing at time point 2 (*n* = 68).

### Reliability

The RMU for BEA was 0.59, (95% HDCI [0.50, 0.68]), and the sample PSI was 0.57 in the current study. 0.6% had person locations below the lowest item threshold (-2.07 logits), and 0% were above the highest item threshold (2.27 logits). As such, no problematic ceiling or floor effect was found in this sample.

### Item properties and targeting

**Fig. 5:**
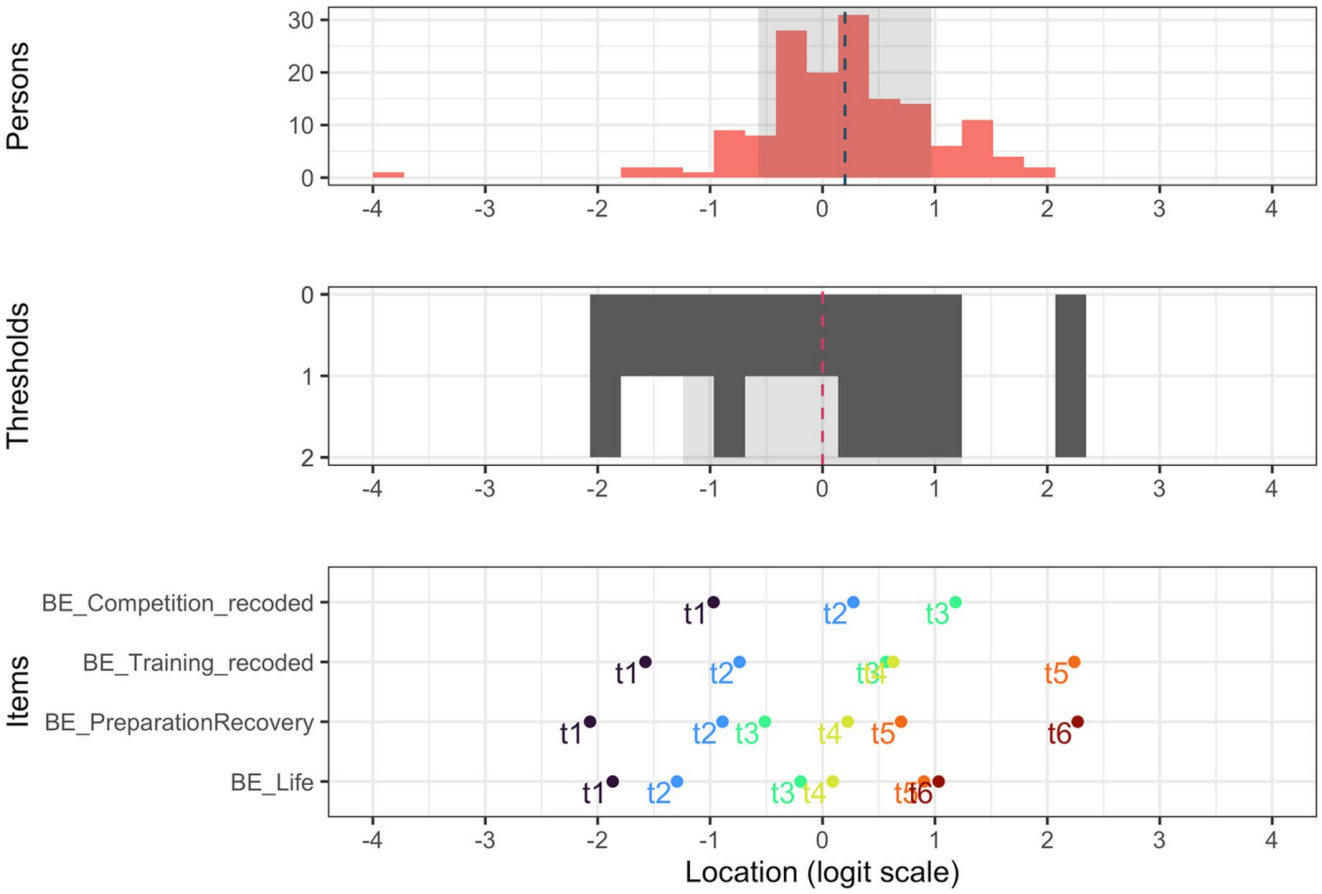
Targeting. Targeting with person, item threshold, and individual item threshold locations on the same logit scale. The top section is a histogram of person locations, and the dotted line is the mean. The middle section is an inverted histogram of item thresholds, and the dotted line is the mean. The bottom section is the threshold location for each individual item. All sections use the same logit scale on the x-axis.

Item thresholds for each item are presented in Table 3. When placing item thresholds and person locations on the same logit scale, BEA items fit (target) respondents well, with a mean person location of 0.2 (SD = 0.77) and a mean item threshold location of 0 (SD = 1.24). See targeting in Fig. 5.

**Table 3:** Item thresholds (ordered).

Item	Threshold						Item location
	1	2	3	4	5	6	
BE_Competition_recoded	-0.97	0.28	1.18				0.16
BE_Training_recoded	-1.58	-0.74	0.56	0.63	2.24		0.22
BE_PreparationRecovery	-2.07	-0.89	-0.51	0.22	0.70	2.27	-0.05
BE_Life	-1.87	-1.29	-0.20	0.09	0.90	1.03	-0.22

Note. Item location is the average of item thresholds. Empty cells indicate that the threshold does not exist for the item after response categories were merged.

### Construct validity investigation by correlation

Rasch analyses for PFSS, SWLS, VQ, SMS-2, and CSAI-2R to enable correlations between *θ* values can be found in the online supplementary analysis report. A medium negative association was found between BEA and psychological inflexibility in athletes (PFSS). Medium correlations were found for life satisfaction (SWLS), as well as progress in valued living (VQ progress). A negative medium association was also found with obstruction to valued living (VQ obstruction). Regarding sport motivation, a medium negative association was found between BEA and amotivated regulation. No further significant associations were found for the other sub-scales of SMS-2 (intrinsic, integrated, identified, introjected, and external). For performance anxiety and the CSAI-2R sub-scales, a medium negative association was found for cognitive anxiety, and a medium positive association was found for self-confidence. No significant correlation was demonstrated with somatic anxiety. Correlation results between BEA and the other scales are presented in Table 4.

In the partial correlations, the associations between BEA and the VQ sub-scales (progress and obstruction) somewhat decreased but were still of medium size. The negative medium correlation with amotivated regulation was still medium in the partial correlation. The medium association with self-confidence dropped and became small, and the medium negative association with cognitive anxiety also dropped and was no longer significant. Sensitivity analyses with reduced sample sizes were not performed for the partial correlations, as the results from the zero-order correlations did not indicate it to be necessary. The partial correlations are presented in Table 5.

BEA and the other scales were not completed on the same day as intended for all participants. The majority of respondents completed BEA and the other scales within 24 h (*n* = 104, 85.25%), while the remaining 17 (13.93%) varied in delay between 1 and 21 days. Sensitivity analyses for the zero-ordered correlations were therefore conducted to compare correlations between within-24-hour completers and the full sample of survey completers. One case of significant difference was found, which was for the association between amotivated regulation (a sub-scale in SMS-2) and BEA, although both correlations were still in the same interpretative range with confidence intervals that overlapped to a large degree (*r* = − .35, *p* < .001, 95% CI [-0.50, − 0.19], *n* = 120; *r* = − .35, *p* < .001, 95% CI [-0.51, − 0.17], *n* = 103), the result of the sensitivity analysis with reduced sample size was therefore not used.

**Table 4:** Correlations between BEA and other scales.

Scale	Correlation parameter	*r*	rho	*p*	95% CI	*N*
PFSS	*θ*	− 0.35*		< 0.001	− 0.50, − 0.18	120
SWLS	*θ*	0.48*		< 0.001	0.33, 0.61	120
**VQ**						
Progress	*θ*	0.48*		< 0.001	0.33, 0.61	120
Obstruction	*θ*	− 0.47*		< 0.001	− 0.60, − 0.32	120
**SMS-2**						
Intrinsic	*θ*	0.16		0.077	− 0.02, 0.33	120
Integrated	*θ*	− 0.006		0.949	− 0.18, 0.17	120
Identified	*θ*	0.04		0.697	− 0.14, 0.21	120
Introjected	*θ*	− 0.01		0.878	− 0.19, 0.17	120
External	*θ*	− 0.05		0.580	− 0.23, 0.13	120
Amotivated	*θ*	− 0.35*		< 0.001	− 0.50, − 0.19	120
**CSAI-2R**						
Cognitive anxiety	*θ*	− 0.32*		< 0.001	− 0.47, − 0.15	120
Somatic anxiety	*θ*	− 0.10		0.296	− 0.27, 0.08	120
Self-confidence	*θ*	0.37*		< 0.001	0.20, 0.51	120
Subjective performance rating	Single item ordinal score		0.31*	< 0.001	0.14, 0.47	120

*θ* = theta values (estimated with Weighted Likelihood Estimation); *r* = Pearson’s product-moment correlation; rho = Spearman’s rank correlation; * = *p* < .05; CI = confidence interval; N = number of participants; PFSS = Psychological Flexibility in Sport Scale; SWLS = Satisfaction With Life Scale; VQ = Valuing Questionnaire; SMS-2 = Sport Motivation Scale-2; CSAI-2R = Competitive State Anxiety Inventory-2 Revised.

**Table 5:** Partial correlations between BEA and sub-scales of VQ, SMS-2, and CSAI-2R.

Scale	Correlation parameter	*r*	*p*	95% CI	*N*
**VQ**					
Progress	*θ*	0.34*	< 0.001	0.17, 0.49	120
Obstruction	*θ*	− 0.33*	< 0.001	− 0.48, − 0.16	120
**SMS-2**					
Intrinsic	*θ*	0.13	0.750	− 0.05, 0.30	120
Integrated	*θ*	− 0.12	0.753	− 0.29, 0.06	120
Identified	*θ*	− 0.005	0.959	− 0.18, 0.17	120
Introjected	*θ*	0.07	0.929	− 0.11, 0.24	120
External	*θ*	0.09	0.921	− 0.09, 0.27	120
Amotivated	*θ*	− 0.36*	< 0.001	− 0.50, − 0.19	120
**CSAI-2R**					
Cognitive anxiety	*θ*	− 0.15	0.219	− 0.32, 0.03	120
Somatic anxiety	*θ*	0.08	0.372	− 0.10, 0.26	120
Self-confidence	*θ*	0.23*	0.035	0.05, 0.39	120

Partial correlations control for shared variance among sub-scales within each scale; *θ* = theta values (estimated with Weighted Likelihood Estimation); r = Pearson’s product-moment partial correlation coefficient; * = *p* < .05; CI = confidence interval; N = number of participants; VQ = Valuing Questionnaire; SMS-2 = Sport Motivation Scale-2; CSAI-2R = Competitive State Anxiety Inventory-2 Revised.

## Discussion

The purpose of the current study was to develop and evaluate BEA, a sport psychology instrument to measure and do therapeutic work with values and values-based behavior in athletes. Rasch analysis was used to investigate its psychometric properties, and construct validity was also investigated by correlation. BEA did initially demonstrate satisfactory scale dimensionality, although item thresholds were disordered for two items (BE_Competition and BE_Training). After merging some of the response categories in those two items, an overall satisfactory model fit for a unidimensional scale with locally independent items and ordered response categories was provided. BE_Life was close to the upper threshold in the conditional item infit investigation while also demonstrating a deviating loading on the first residual contrast, indicating it as potentially underfit to the model. However, as item infit was within the simulated critical values and the item-restscore did indicate misfit, the item was kept. The small sample size is a limitation in detecting item misfit using both infit and item-restscore statistics^38^. BE_Life is conceptually focusing on another part of a proposed values-dimension, life outside of sports, compared to the other three items that focus on values for sport participation itself. From a validity perspective, it is not unexpected to see the item deviate a bit from the other items, although all four items still seem to fit within a general values dimension for the athletes and to be used together. However, this should be considered and further evaluated in future psychometric evaluations of the instrument, where a larger sample size would be a recommended priority. In therapeutic work, it is not uncommon to focus on separate topics during a session and divide some work into each value domain (item), to facilitate the work with behavior change in relation to each value. This is a common way of working with values, by selecting and choosing to work with certain values or life domains one at a time^5,55^. It should therefore be noted that the current study does not primarily investigate the therapeutic utility of using BEA in various practical ways with individuals, but more specifically, whether the four items seem to measure values in athletes in a coherent way. The alignment of intervention content and measurement should therefore be considered when using BEA in upcoming clinical and research use, as well as the psychometric properties in that (future) specific context. No significant DIF was found for either demographic (sex, age) or sport variables (competitive level, sport type, current injury). The test-retest investigation also supported item properties’ stability over time using a DIF framework. A limitation of the test-retest investigation was the variability in time for the retest-taking of BEA (median = 16.80 days; IQR = 6.18 days). The increased response time could potentially have opened up for an increased probability of ‘true’ change in the latent construct, although the DIF framework should be more robust towards such bias than traditional test-retest correlation. However, a standardized retest timepoint would have been favorable. In general, DIF results should be interpreted cautiously since the limited sample size of the compared sub-groups decreases the probability of detecting significant statistical differences in item functioning.

RMU was used to investigate reliability and was estimated in this sample at 0.59 (95% HDCI, 0.50, 0.68) for BEA, which does not indicate strong reliability and is a reason for cautious interpretation of the performance of the scale. The RMU was considerably lower than for some of the other scales in this study, which were in the range of 0.72 to 0.90, except for some of the sub-scales in SMS-2 that ranged between 0.25 and 0.75 (see the Methods section for each scale’s RMU). One possible reason for the relatively low RMU of BEA is that it consists of only four items, which can affect the RMU since less information is available to estimate reliability. The merging of response categories also reduces the precision of the BEA. Additionally, all reliability metrics are affected by the sample’s latent variable variation, which is quite narrow in our sample at SD = 0.77. Therefore, BEA response data was also simulated with SD = 1 and resulted in RMU = 0.68, showing how sample-based reliability estimates can be affected by sample characteristics. However, the limited reliability should be acknowledged and calls for caution in practical use, both in clinical and research settings, and further research is warranted. Developing and evaluating a reworked set of response categories/labels and collecting data from another, larger sample may result in improved reliability.

BEA was not validated against another instrument that measures sport-related values or values-based behavior in sport. Therefore, a criterion validity investigation was not possible. However, construct validity by correlating scales that are partly related was investigated. An overall caution regarding the construct validity correlations is that the scales that underwent Rasch analysis (PFSS, SWLS, VQ_progress, and all sub-scales of both SMS-2 and CSAI-2R) resulted in adjusted versions of the scales (Rasch analyses are found in the online supplementary analysis report).

A medium negative association with sport-related psychological inflexibility was found. Support of the medium-sized discriminant validity is partly in line with ACT theory that underpins both BEA and PFSS. It should be noted that PFSS does not cover all the processes that constitute psychological flexibility/inflexibility; instead, items primarily focus on being obstructed by thoughts and feelings (psychological inflexibility and experiential avoidance), rather than focusing on values and values-based behavior^20^. This is a plausible explanation for not finding a stronger association with BEA. Convergent validity for life satisfaction was also supported by a significant medium correlation (with SWLS), which is consistent with ACT theory in the sense that pursuing values is expected to promote a satisfied and healthy life^13^. The current results suggest that it could be the case for sport-related values pursuit for athletes as well. BEA was found to be associated with valued living in general to some extent, showing a medium correlation with progress in valued living (VQ_progress), and a medium negative correlation with obstruction to valued living (VQ_obstruction). These results support that BEA measures aspects of values-based behavior which should be expected, although BEA targets other value domains (sport-related) that are not covered in general measures of valued living. Partial correlations between BEA and the VQ sub-scales decreased but were still in the medium range, suggesting that both VQ sub-scales contribute with unique information of a values dimension, with distinct associations to BEA.

Discriminant validity between BEA and performance anxiety was supported and demonstrated by a medium negative correlation with cognitive anxiety. Further, a medium positive correlation was also demonstrated with self-confidence. This is theoretically consistent with values-based behavioral patterns that are more likely to be under appetitive rather than aversive emotional control^13^. High levels of anxiety paired with values-inconsistent behavior could be an indication of the latter. However, the partial correlations decreased and were reduced from medium to small size for self-confidence, and were non-significant for cognitive anxiety. This indicates that the relationships found in the zero-ordered correlations to some extent depended on shared variance among the sub-scales of CSAI-2R, weakening the case for distinct sub-dimensional associations with performance anxiety.

Regarding sport motivation, a negative association of medium magnitude was found between BEA and amotivated regulation. The partial correlation was also medium and close to the zero-order correlation, and the confidence intervals overlapped to a large degree. This suggests that it is a distinct dimension of sport motivation associated with BEA. The amotivated regulation items focus on whether to continue sports at all. No further sub-scales of SMS-2 were associated with BEA. This was unexpected since values, from a behavioral theory perspective, can be described as having motivating functions for values-based behavior. Values-based behavior is also often characterized as being under appetitive emotional control and intrinsically engaging^13^. It is therefore, to some extent, theoretically consistent with aspects of self-determination theory (the motivational theory that primarily underlines SMS-2), such as *autonomy*, which can be achieved when behavior is volitional and/or an authentic expression of self, and in line with personal values^27,56^. Some items in SMS-2 are formulated to explore quite harsh or extreme experiences in relation to sport, such as “Because people I care about would be upset with me if I didn’t [practice my sport]”. Some of these types of items were also less endorsed, resulting in quite skewed response category distributions for some items in the current sample of adult elite athletes. Associations between BEA and sub-scales of SMS-2 could possibly be sample sensitive and should therefore be examined in further sports contexts. The factor structure of the Swedish translation of SMS-2 has not previously been confirmed (e.g., via CFA) in this context (Swedish elite athletes), which should be taken into account when interpreting the results. In the current study, dimensionality and psychometric properties of the SMS-2 sub-scales were evaluated using Rasch analysis (see the online supplementary analysis report). The low reliability for some of the sub-scales, especially for introjected regulation (RMU = 0.25), external regulation (RMU = 0.61), and integrated regulation (RMU = 0.63), should be acknowledged when interpreting the correlations between BEA and SMS-2. A limitation regarding SMS-2 was the current suggested six-factor solution with only three items per sub-scale^27^, for which a full-step Rasch analysis was difficult to conduct. Some items performed poorly but were kept in each sub-scale since subsequent Rasch analyses with only two items often are limited. For that, a larger sample size would have been required, together with multiple response categories that function properly to uphold reliability.

Convergent validity between BEA and the subjective performance rating was demonstrated to some extent with a medium correlation. This was expected as an instrument measuring values consistent behavior that includes performance events (e.g., the competition and training items) reasonably should reflect how the athletes are experiencing their performance outcome to some degree. Since this correlation was based on ordinal scores and a single-item measure, interpretations should be made cautiously.

BEA adds to a short list of available values measures in sport. Besides MSQ, which measures general aspects of meaning and purpose in athletes in the form of a traditional scale^11^, the format of BEA enables its use as a therapeutic tool as well. BEA includes not only ratings of behavioral commitment to values but also the process of qualitatively identifying values (related to each item), as well as the identification and ratings of obstacles and forming a behavioral plan to pursue chosen values.

It is notable that all scales with seven response categories had disordered item thresholds and needed to be recoded in this study. This is in line with previous findings that scales with (too) many response categories may not function properly^24,25^. The relatively small sample size is a limitation of the current study, which impacts the analysis of dimensionality, as well as item parameter estimation and DIF analyses^36^. Most of the sample was team-sport athletes (*n* = 136, 88%) over individual sport athletes (*n* = 19, 12%). It not only makes it difficult to detect potential DIF between these groups of athletes, but also this imbalance in sport types means that the instrument was primarily evaluated for team sport athletes rather than individual sport athletes. This limits the generalization of how BEA works in various sports populations. Future studies should be conducted in various sports contexts with not only larger sample sizes but also in more diverse samples. Another limitation is that criterion validity with another sport-related values instrument was not conducted and should be investigated. Associations between BEA and objective performance measures would also add insight into the relationship between values work with athletes and performance outcomes. BEA should also be investigated in future research exploring how it functions in a longitudinal design, such as an intervention study measuring behavioral change.

### Practical implications

For the future use of BEA, it may be advisable to reduce the number of response categories. In this study, this was suggested for two items: the competition item (four useful response categories; combine 1 + 2, 3 + 4, and 5 + 6) and the training item (six useful response categories; combine 1 + 2). It could also be useful to add labels to each response category, which may ease response category interpretation for respondents. Since these suggestions are based on a single psychometric evaluation and differ among items, further validation of the response categories is needed prior to a definitive recommendation.

## Conclusions

BEA showed satisfactory psychometric properties in general as a unidimensional scale of measuring behavioral commitment to values in athletes. BEA also offers a therapeutic tool to work with values and behavioral change in sport. Items were locally independent. Further, after merging response categories for two items (competition and training), item thresholds were ordered, and response category monotonicity was reached. Labeling the response categories in BEA could be a future potential improvement to investigate. No indication of invariance was found through differential item functioning (DIF) analyses, but should be interpreted cautiously due to the small sample sizes of the compared sub-groups. Construct validity investigations by correlation were supported with associations found between BEA and psychological inflexibility, general values, life satisfaction, subjective performance, and subtypes of performance anxiety and sport motivation. BEA should be further evaluated with larger samples in various sports contexts. BEA should also be investigated in longitudinal designs as a measure of behavioral change in athletes.

## Supplementary Information

Below is the link to the electronic supplementary material.

Supplementary Material 1

Supplementary Material 2

## Data Availability

The datasets used and analyzed during the current study are available from the corresponding author upon reasonable request. All code and analyses are available in an online supplementary analysis report: https://gustafre.github.io/Bulls-Eye-for-Athletes/. The Bull’s-Eye for Athletes instrument is presented in Supplementary file 1 (Swedish version) and 2 (English version).
